# Measles vaccination perceptions and willingness to consider novel vaccination approaches in Cambodia’s floating villages

**DOI:** 10.1128/jvi.01718-25

**Published:** 2026-01-07

**Authors:** Benjamin L. Sievers, Sudipta Hyder, Malen Chan, Sowath Ly, Ly Sovann, Erik A. Karlsson

**Affiliations:** 1 Virology Unit, Institut Pasteur du Cambodge 533891, Phnom Penh, Cambodia; 2 Department of Medicine, University of Cambridge 2152, Cambridge, United Kingdom; 3 Infectious Disease Unit, Columbia University Irving Medical Center 21611, New York, New York, USA; 4 Epidemiology Unit, Institut Pasteur du Cambodge 533891, Phnom Penh, Cambodia; 5 Communicable Disease Control Department, Ministry of Health 301685, Phnom Penh, Cambodia; University of Kentucky College of Medicine, Lexington, Kentucky, USA

**Keywords:** vaccination, vaccination attitudes, measles, self-vaccination, dry-powder inhalation

## Abstract

Globally, the measles virus is making a comeback. Measles still claims the lives of over 100,000 children annually and remains a leading cause of preventable child mortality. Despite the availability of a highly effective and safe measles vaccine since the late 1960s, challenges in vaccine uptake persist. In 2019, Cambodia experienced a resurgence of measles cases, leading to the loss of its elimination status. Many of these cases were reported in geographically isolated and highly mobile communities, such as the floating villages along the Mekong Delta, including Prek Toal. Due to their mobility and geographical isolation, these villages face unique barriers to routine healthcare access and were directly impacted by the 2019 outbreak. To assess vaccination attitudes in this unique population, the Vaccine Attitudes Examination (VAX) scale was adapted to include specific questions about self-vaccination for measles. The modified survey was translated into Khmer and administered to families or caretakers with children living in the floating villages of Prek Toal. The survey results demonstrated broad support for vaccinations among the Prek Toal community, with most respondents expressing confidence in the safety and effectiveness of vaccines. Additionally, while families showed openness to the concept of self-vaccination, there was a strong preference for vaccines to be administered by healthcare professionals. These findings highlight the positive attitudes toward vaccination within this mobile and geographically isolated population, as well as their willingness to consider novel approaches like self-vaccination. Addressing logistical challenges and preferences for healthcare-administered vaccines will be critical in enhancing vaccination strategies in such hard-to-reach communities, ultimately aiding in the fight against measles resurgence.

**IMPORTANCE:**

Measles is one of the most transmissible human viruses, and sustaining high vaccination coverage is essential for preventing its resurgence. Remote and mobile communities often have limited, intermittent access to routine health services, yet little is known about their perspectives on vaccination or their openness to new delivery approaches. Cambodia’s floating villages represent one such setting, where seasonal changes, mobility, and geographical distance can make routine vaccination more difficult to access. In this study, we examined parental attitudes toward measles vaccination in a floating village and assessed interest in simplified, needle-free vaccination methods. We found high confidence in vaccines and healthcare providers, along with a willingness to consider alternative delivery formats if they are safe and easy to use. These results suggest that logistical challenges rather than hesitancy are key contributors to immunity gaps.

## INTRODUCTION

Measles remains a significant global health threat, despite the availability of a highly effective vaccine for nearly 50 years. Between 2023 and 2025, unprecedented outbreaks have occurred worldwide, including fatal outbreaks in Yemen and the United States, highlighting the persistent challenge of controlling this highly transmissible virus (1, 2). As one of the most contagious pathogens ever discovered, measles requires exceptionally high levels of population immunity to prevent its spread. While global vaccination efforts have drastically reduced measles incidence and led to elimination in several countries, measles continues to claim the lives of children, particularly in regions without centralized, active vaccination services. Indeed, the disease remains endemic in hotspots such as Ukraine, the Democratic Republic of the Congo, and Mongolia (3, 4). The Western Pacific Region, once a leader in measles control, exemplifies this struggle: both Mongolia and Cambodia achieved elimination in 2014 and 2015, respectively, but disruptions in vaccination campaigns, case importation, and persistent pockets of unvaccinated individuals have led to a resurgence of the disease (5).

Cambodia is a least developed country in Southeast Asia. Prek Toal is a floating community in Battambang Province in northern Cambodia. Tucked away at the top of the Tonlé Sap Biosphere Reserve, Prek Toal is home to 13,823 people. The Prek Toal region is renowned for its rich biodiversity, particularly its flooded forests and bird sanctuary, which attract a wide variety of wildlife, including migratory birds (6). While this extensive biodiversity supports the livelihoods of local communities through fishing and farming, it also increases the risk of zoonotic and infectious diseases (7). Additionally, the seasonal flooding that expands the lake and the constant movement of the floating villages further complicates disease control and healthcare access, making communities like Prek Toal particularly vulnerable to infectious diseases (8).

Cambodia has implemented a highly successful measles program, achieving official elimination in 2015; however, due to pandemic-related vaccination campaign delays and cancellations, measles could reemerge in harder-to-reach communities, including the floating communities on the Tonlé Sap (9, 10). In 2019, 676 measles cases were confirmed in Cambodia, many of which occurred in the floating villages (11). More recently, in 2025, Cambodia experienced 375 measles cases in 2024 and 2,150 by April 2025 (12, 13). According to WHO/UNICEF Estimates of National Immunization Coverage, Cambodia’s first and second doses of a measles-containing vaccine in 2023 were 79% and 64%, respectively (14). During the COVID-19 pandemic, Cambodia had a similar vaccine rollout strategy to many other nations, with both centralized and mobile rollout of COVID-19 vaccines (15). As most Cambodians are vaccinated for measles at centralized hospitals and health centers as per the national standard immunization schedule, highly mobile communities, including boat-dwelling communities, are less likely to visit these health centers and can remain unvaccinated (10). High vaccination rates are required to eradicate measles, including in harder-to-reach communities.

As the elimination of measles and, ultimately, the global eradication of measles require high levels of measles immunity, strategies must be developed to reach communities most distant from centralized vaccination centers. Many vaccination approaches, such as microneedle patches and inhalable vaccines, make methods such as self-vaccination or minimal-training vaccination amenable (16–20). However, to gage the feasibility of these orthogonal approaches, general vaccination attitudes become highly relevant. To understand the general attitudes toward vaccinations in hard-to-reach and remote communities, we surveyed vaccination attitudes in the floating villages of Prek Toal.

## MATERIALS AND METHODS

### Questionnaire development

The Vaccination Attitudes Examination (VAX) scale is a standardized questionnaire set that provides a method for identifying vaccination resistance and general vaccination attitudes (21). The VAX scale includes 12 questions with a strongly disagree and strongly agree (1–6) rating scale and was expanded to include 4 relevant questions on measles self-vaccination. Ratings can be associated with broader concepts such as “Unforeseen Problems” or “Natural Exposure.” All questionnaires and documents were translated into Khmer. All English and Khmer documents are available upon request.

### Population and sampling

**Fig 1:**
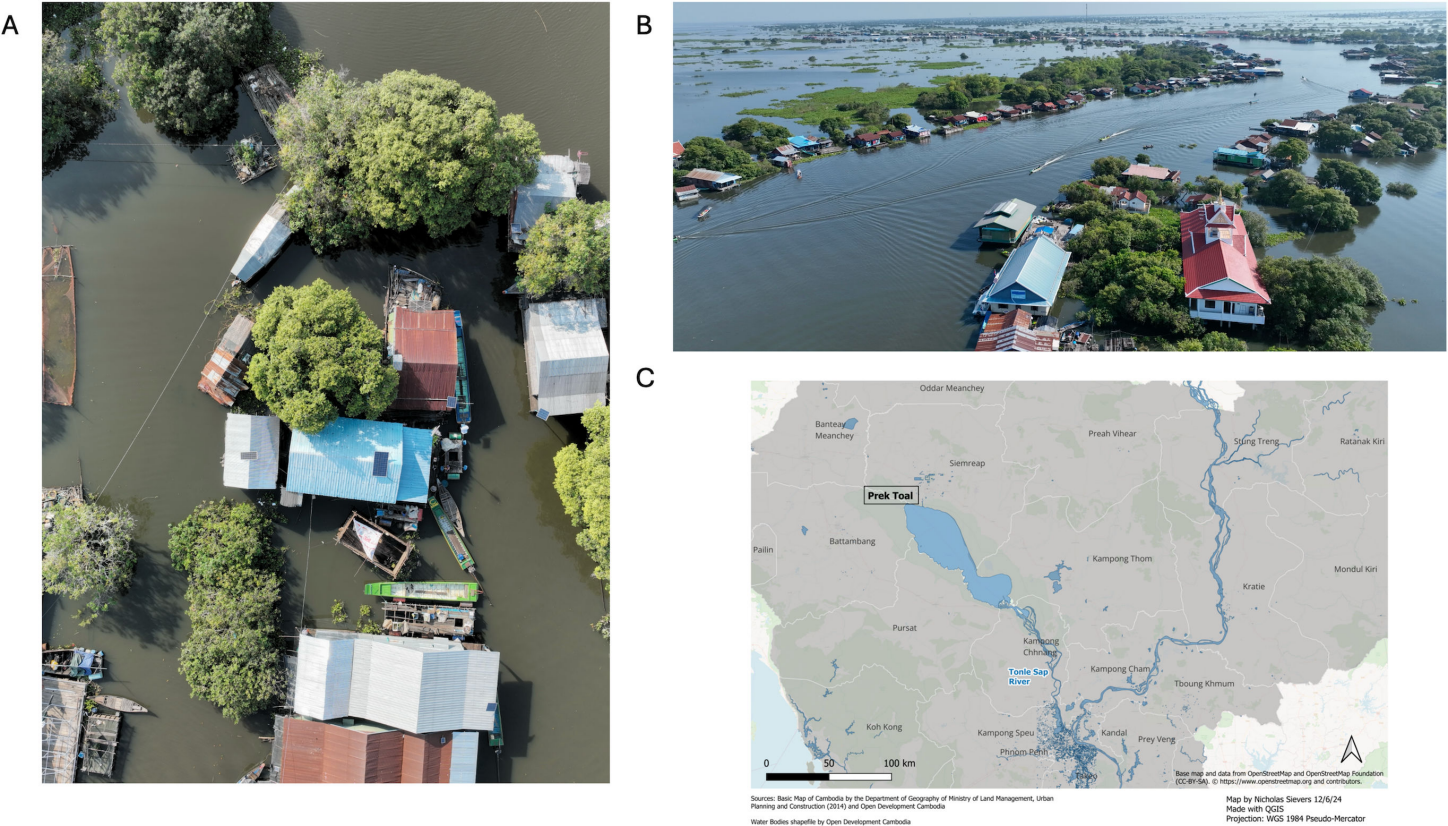
(**A**) Aerial view of a collection of homes in Prek Toal, Cambodia. (**B**) Lateral view of more homes in Prek Toal, Cambodia. (**C**) Location of the area sampled in Cambodia. The map was generated using QGIS by Nicholas Sievers. Photographs were taken by Benjamin L. Sievers.

**Fig 2:**
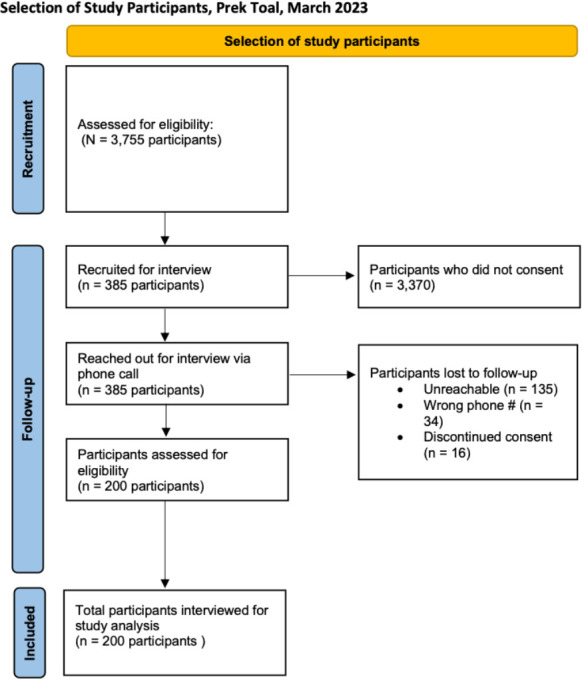
Selection of study participants, Prek Toal, March 2023.

This study sought parents and/or guardians with one or more children living within the Prek Toal community (Fig. 1). A list of parents and/or caretakers who fit our inclusion criteria was provided by the Battambang Provincial Health Department. This list consisted of a total of 385 individuals who were contacted in the recruitment for this study. Interviewers were Cambodian researchers and conducted the interviews in Khmer. Of the 385 contacted, 135 (35%) were unreachable, 34 (9%) had the wrong contact number, and 16 (4%) refused. Ultimately, 200 respondents were included in the study (Fig. 2).

### Statistical analysis

All statistical analyses were conducted using R Studio IDE. A six-point Likert scale was employed to analyze the survey data. Responses were coded and quantified accordingly, facilitating the assessment of participant attitudes and perceptions. Descriptive statistics, including frequency, means, and standard deviations, were calculated to summarize the central tendencies and variabilities of the responses. Additionally, inferential statistical methods, such as the chi-squared test of independence, were applied where appropriate to determine significant differences between variables.

## RESULTS

### Participants

The survey respondents included 200 parents or guardians with at least one child from the Prek Toal community. Among the 200 respondents, 62.5% were women, and 37.5% were men, with an overall mean age of 36.3. Eleven percent were guardians of children. Educational backgrounds varied, with 40% having completed primary education, 35% secondary education, and 25% having no formal education. General demographics and other characteristics can be found in Table 1.

**TABLE 1:** Demographic characteristics and descriptive information of surveyed participants

Characteristic	No. of participants (*n*) (*N* = 200)	% of participants (*n*%)
Gender identity		
Male	75	37.5
Female	125	62.5
Age (years)		
<18	0	0
18–28	42	21
29–38	87	43.5
39–48	39	19.5
49–58	20	10
59–68	10	5
69+	2	1
Highest level of education		
None	20	10
Non-formal education	1	0.5
Primary	91	45.5
Secondary	58	29
Degree level/high school	29	14.5
College/university and above	1	0.5
Number of children		
0	22	11
1–2	68	34
3–4	69	34.5
5–6	32	16
7–8	7	3.5
9–10	1	0.5
11+	1	0.5
Received COVID-19 vaccine		
Yes	190	95
No	10	5

### General attitudes toward vaccination

**Fig 3:**
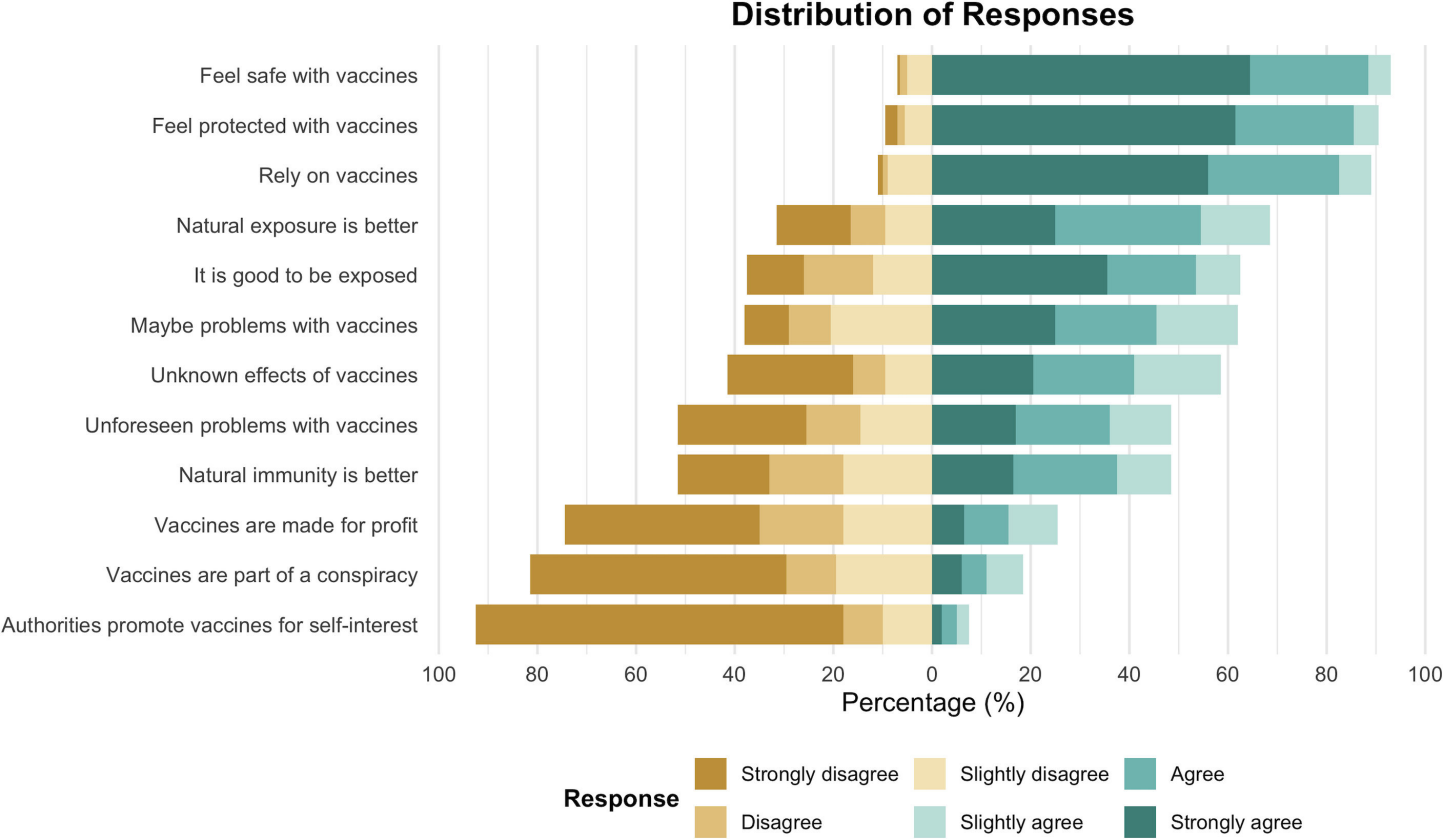
Attitudes toward measles vaccination. Responses to questions from the VAX scale were measured using a six-point Likert scale ranging from “Strongly disagree” (1) to “Strongly agree” (6). Each item corresponds to a broader conceptual domain associated with the VAX scale.

The community displayed a broadly positive attitude toward vaccinations (Fig. 3). Most respondents (*n* = 186; 93%) agreed that vaccines are safe, and 90% (*n* = 180) believed they are effective in preventing diseases. While 58% of respondents did express some concerns about the potential side effects of vaccines, these concerns did not significantly diminish overall support for vaccination. Opinions were more evenly split regarding the belief that natural immunity lasts longer than vaccine-induced immunity, with 52% disagreeing versus 48% agreeing. Notably, 92% of participants strongly rejected the idea that vaccines are designed for financial gain rather than public health protection, indicating a high level of trust in the intentions behind vaccination programs (Fig. 3).

### Knowledge and awareness of measles vaccinations

Most respondents (95.5%) were familiar with the measles virus, and 86% had children who received a measles-containing vaccine.

### Self-vaccination attitudes

When asked about the possibility of self-vaccination for measles, 89% of respondents expressed willingness to administer vaccines to their children themselves, if provided with proper instructions and supplies. However, 92% indicated that they preferred to have a healthcare professional administer the vaccines (Table 2).

**TABLE 2:** Measles vaccination-specific questions

Question	No. of participants (*n*) (*N* = 200)	% of participants (*n*%)
Are you familiar with the measles virus?		
Yes	191	95.5
No	9	4.5
Are your children vaccinated for measles?		
Yes	172	86
No	28	14
If vaccination did not require an injection, would you like to give it to your child yourself?		
Yes	178	89
No	22	11
Would you prefer to have a healthcare worker administer it?		
Yes	184	92
No	16	8

## DISCUSSION

Parents from the floating villages of Prek Toal, Cambodia, demonstrated broadly positive attitudes toward vaccinations, viewing them as safe and essential for protecting health rather than as tools for financial gain. While most respondents preferred vaccines to be administered by healthcare workers, many were open to the idea of self-administering a simple-to-use measles vaccine to their children, highlighting a receptiveness to decentralized vaccination strategies. Though some concerns about potential side effects of vaccines were raised, these concerns did not significantly diminish overall support for vaccination. These findings highlight the potential for exploring self-administered or community-administered vaccination methods in areas with limited healthcare access, though they also underscore strong trust placed in healthcare providers.

Vaccination remains one of the most effective tools for controlling measles outbreaks and preventing child mortality, yet global vaccination coverage remains insufficient (22). Globally, reported measles cases increased from 170,000 in 2022 to over 320,000 in 2023, largely due to COVID-19-related immunization disruptions (14). Even more recently, there have been over 800 confirmed measles cases in the United States, with three deaths (1). In Cambodia, measles vaccination efforts made significant progress in the years prior to the COVID-19 pandemic, but global disruptions to health services during the pandemic likely affected measles vaccination progress. Between January and April 2025, Cambodia reported 2,150 cases of measles, significantly higher than the 375 confirmed cases in all of 2024 (23, 24).

The persistent struggle with measles and ongoing challenges in vaccination efforts in Cambodia and globally underscore the urgent need for alternative strategies to achieve eradication, a goal that, while debated, remains attainable. Advances in vaccine delivery technologies have made self-administration a practical possibility. Innovative sharps-free methods, such as microneedle patches and inhalable vaccines, simplify administration and make vaccination more accessible (17, 19). For example, a recent clinical trial in Gambia demonstrated that measles and rubella microneedle patch vaccines were highly safe, well tolerated, and immunogenic in children, offering a promising alternative to traditional subcutaneous vaccines (25). Other studies have demonstrated the feasibility of dry-powder inhalable measles vaccines, which are thermostable and may be amenable to self-vaccination (20, 26, 27). Additionally, an aerosolized measles vaccine was shown to be well tolerated and immunogenic in children (26, 28).

Regardless of delivery mechanisms, opinions and knowledge toward vaccinations are important in raising the vaccination coverage level. In our findings, nearly 62% of respondents indicated that they agreed with the phrase “natural exposure to viruses and germs gives the safest protection,” suggesting that some individuals hold concurrent beliefs on natural infection and vaccination. Shifting opinions toward vaccination have become a significant global health concern in recent years. In Zimbabwe in 2022, over 700 children died of measles infections during an outbreak largely fueled by undervaccination due to rumors and misinformation circulating in the media (29). Focusing on methods of vaccination that bring more power and autonomy to communities can increase people’s confidence in vaccination. No children should die from preventable diseases.

The rapid resurgence of measles underscores the urgent need for targeted and orthogonal vaccination strategies, particularly in mobile and hard-to-reach communities like the floating villages of Prek Toal. Low vaccination coverage can be due to many different logistical challenges, such as environmental conditions or vaccine cold chain constraints, and this study demonstrates that despite these conditions, these communities exhibit a strong overall confidence in vaccines and their efficacy, as well as an openness to innovative approaches like self-vaccination. However, the preference for healthcare-administered vaccines reflects the importance of trusted healthcare delivery systems. Importantly, a large proportion of the Prek Toal community, nearly 35% of those contacted, were unable to respond to our survey and may represent an even more geographically isolated community. Efforts to improve vaccine access must address these logistical barriers while respecting community preferences, paving the way for more effective and inclusive vaccination campaigns. Strengthening these initiatives in geographically isolated populations is essential for regaining measles elimination status and preventing future outbreaks.

Taken together, these findings demonstrate a critical opportunity to reimagine measles vaccination strategies in response to a global resurgence. Self-administration platforms such as inhalable or microarray vaccines create another avenue for extending the reach of immunization efforts, especially in settings where conventional delivery is limited. Yet, our findings demonstrate that innovation must be paired with trust, as community engagement, transparent information, cultural sensitivity, and respect for local preferences must be essential in designing and developing methods to increase vaccination coverage. By aligning vaccination technologies and approaches with feedback and experience from parental perspectives, particularly in hard-to-reach communities, we can improve access to life-saving vaccinations.

### Implications for future interventions

Overall, the findings highlight strongly supportive attitudes toward vaccination within the floating villages of Prek Toal, along with a willingness to consider self-vaccination, if adequate training and resources are provided. This presents an opportunity to enhance vaccine uptake through community-based interventions and educational programs that empower parents to vaccinate their children. However, the preference for professional administration underscores the need for continued investment in healthcare infrastructure and outreach to ensure vaccines remain accessible and are administered safely.

### Key messages

#### What is already known on this topic

Measles remains a leading cause of preventable child mortality globally, particularly in hard-to-reach and mobile communities. Despite the long-standing availability of an effective vaccine, vaccination coverage in remote areas, such as Cambodia’s floating villages, remains insufficient due to barriers like geographical isolation and limited access to healthcare services. Novel approaches, such as self-vaccination, have been proposed to improve vaccine delivery in these settings.

#### What this study adds

This study highlights that parents in Cambodia’s floating villages generally have positive attitudes toward measles vaccination and exhibit a willingness to consider self-vaccination methods if adequate resources and training are provided. However, there remains a strong preference for vaccines to be administered by healthcare professionals, emphasizing trust in the healthcare system.

#### How this study might affect research, practice, or policy

The findings suggest that innovative vaccine delivery methods, such as self-vaccination, could be viable in remote communities if paired with robust education and resource provision. This study underscores the need for targeted strategies to address logistical barriers in hard-to-reach areas and calls for policy and programmatic efforts to integrate community-based vaccination initiatives with healthcare outreach.

## Data Availability

All data supporting the findings of this study are included in the paper. De-identified survey data sets and study instruments (English and Khmer versions) are available from the corresponding author upon reasonable request, subject to approval from the Cambodian National Ethics Committee for Health Research and in accordance with participant confidentiality requirements. No sequencing data, proprietary data sets, or restricted-access materials were generated for this study.
